# Supplementary material for: Cultural adaptation and validation of the short food literacy questionnaire (SFLQ) for adults in Lebanon
Source: PLoS One. 2025 Oct 9;20(10):e0334137. doi: 10.1371/journal.pone.0334137 (PMC12510489; doi:10.1371/journal.pone.0334137)
Supplement: Attachment — Submitted filename: Comments to Authors22.docx [file pone.0334137.s002.docx]

**Comments to authors**

This manuscript addresses an important and timely topic: the cultural adaptation and validation of the Short Food Literacy Questionnaire (SFLQ) for use among Arabic-speaking adults in Lebanon.

However, several aspects need improvement:

1. **Abstract:** The abstract is generally strong but could be improved through minor rewording and slight elaboration in the methods and results section.
2. **Introduction:**
i. The section is **too long and text-heavy**, Consider breaking long paragraphs and summarizing repetitive points to improve readability.
ii. Some sentences are grammatically awkward or wordy. A light language polish would greatly enhance clarity. Eg There was 69 million undernourished individuals… better to say were instead of was
iii. Some statements are repeated across paragraphs. Eliminate duplication to tighten the narrative.
iv. The final paragraph begins with a lengthy description of past studies and only introduces this study’s aim toward the very end. Reverse this order: start with the study aim, then explain why this aim is relevant.
3. **Method:**
i. Criteria: Exclusion of individuals with chronic illness is mentioned but not justified. Why were those with chronic diseases excluded? Clarify.
ii. The translation process needs more elaboration:

- Was it forward-backward translated?

- How many experts reviewed it?

- Was there a pretest or pilot? These are essential steps in cultural adaptation and validation.

iii. Sample size rule is acceptable, but it would be helpful to mention actual achieved power or use Monte Carlo simulation or RMSEA-based calculations, which are more robust for CFA.
iv. Data collection is vaguely described (just says public areas, social media, healthcare settings). Who administered the surveys? Online or face-to-face? Self-filled or assisted? Language of administration? Were there response rate details?
4. **Results**: The Results section is generally clear and well-structured. However, there is a serious discrepancy in the reported mean age, which must be corrected. The CFA and measurement invariance analyses are appropriate and well-reported. Minor improvements in table formatting and interpretation of concurrent validity would further enhance the quality and clarity of the results.

eg The mean age is reported as 14.67 ± 2.94 years, which contradicts the descriptive table (Table 1) that reports 45.07 ± 7.33 years. This major inconsistency must be corrected to reflect the accurate demographic.

In Table 1, the percentage for Primary or less is missing a closing parenthesis: 73 (16.5%. Please correct.

Ensure consistency in the presentation of percentages — either include one decimal place throughout or none.

5. **Discussion:** The Discussion section appropriately interprets the study findings and situates them within existing research. However, it is overly verbose in several areas, with some repetition and dense technical explanation. The authors are encouraged to summarize model fit results more concisely, reconcile unexpected gender differences with prior findings, and expand on the limitations regarding psychometric properties. The clinical and research implications are important and well stated, but would benefit from tighter language. Overall, this section can be significantly improved with focused editing.
6. **Conclusions**
i. Overstatement of "Successful Validation"
ii. Generalization beyond Study Sample: The conclusion appropriately acknowledges the need for broader validation. However, it would be useful to briefly reiterate the limitations of the sample (e.g., online-based, urban bias, higher education level) to ground the claim.
iii. Suggestion for Future Research

**Thank you**
