# Supplementary material for: Cultural adaptation and validation of the short food literacy questionnaire (SFLQ) for adults in Lebanon
Source: PLoS One. 2025 Oct 9;20(10):e0334137. doi: 10.1371/journal.pone.0334137 (PMC12510489; doi:10.1371/journal.pone.0334137)
Supplement: Attachment — Submitted filename: Comments to Authors PONE R1.docx [file pone.0334137.s004.docx]

**Comments to authors**

This manuscript addresses an important and timely topic: the cultural adaptation and validation of the Short Food Literacy Questionnaire (SFLQ) for use among Arabic-speaking adults in Lebanon.

However, several aspects need improvement:

1. **Abstract:** The abstract is generally strong but could be improved through minor rewording and slight elaboration in the methods and results section.

We added few ideas to the methods and we edited the abstract for English language.

2. **Introduction:**
i. The section is **too long and text-heavy**, Consider breaking long paragraphs and summarizing repetitive points to improve readability.

The introduction was reshuffled as advised by the reviewer. We hope it reads better now.

ii. Some sentences are grammatically awkward or wordy. A light language polish would greatly enhance clarity. Eg There was 69 million undernourished individuals… better to say were instead of was

The paper was edited for English language. Thank you.

iii. Some statements are repeated across paragraphs. Eliminate duplication to tighten the narrative.

The introduction was reshuffled as advised by the reviewer. We hope it reads better now.

iv. The final paragraph begins with a lengthy description of past studies and only introduces this study’s aim toward the very end. Reverse this order: start with the study aim, then explain why this aim is relevant.

Corrected as suggested. Thank you.

3. **Method:**
i. Criteria: Exclusion of individuals with chronic illness is mentioned but not justified. Why were those with chronic diseases excluded? Clarify.

We added the following explanation to the methods section:

Individuals with chronic illnesses were excluded because such conditions can significantly influence food choices, eating behaviors, and nutritional requirements, which could influence the assessment of general food literacy.

ii. The translation process needs more elaboration:

- Was it forward-backward translated?

- How many experts reviewed it?

- Was there a pretest or pilot? These are essential steps in cultural adaptation and validation.

We apologize for the oversight. We added these ideas to the methods section:

The scale was forward and backward translated to Arabic by two different translators. The original and translated English versions were then compared by the research team and the two translators to solve any discrepancies. The research team and the translators resolved minor discrepancies. A pilot study was done on 30 adolescents to make sure that all questions are clear to them; no changes were done afterwards.

iii. Sample size rule is acceptable, but it would be helpful to mention actual achieved power or use Monte Carlo simulation or RMSEA-based calculations, which are more robust for CFA.

We added the following paragraph to the methods section:

A Monte-Carlo simulation-based approach described in prior methodological work [40] indicates that, for a one-factor model with 12 items and moderate loadings (0.40-0.70) according to the original validation paper [41], a sample size of approximately 180 is sufficient to achieve stable estimates and adequate power.

iv. Data collection is vaguely described (just says public areas, social media, healthcare settings). Who administered the surveys? Online or face-to-face? Self-filled or assisted? Language of administration? Were there response rate details?

Thank you for this valuable comment. The data collection process included both **face-to-face** and **online** approaches. Surveys were **self-administered** in the **Arabic language**. In public areas and healthcare settings, trained data collectors provided brief instructions and assisted participants when needed, without influencing responses. The online version was distributed through social media platforms using a structured announcement. Due to the combined data collection methods, an exact overall **response rate** could not be determined; however, for the face-to-face recruitment, the **response rate was approximately 78%**. We will clarify these details in the revised manuscript.

4. **Results**: The Results section is generally clear and well-structured. However, there is a serious discrepancy in the reported mean age, which must be corrected. The CFA and measurement invariance analyses are appropriate and well-reported. Minor improvements in table formatting and interpretation of concurrent validity would further enhance the quality and clarity of the results.

eg The mean age is reported as 14.67 ± 2.94 years, which contradicts the descriptive table (Table 1) that reports 45.07 ± 7.33 years. This major inconsistency must be corrected to reflect the accurate demographic.

We apologize for this mistake. It is corrected now.

In Table 1, the percentage for Primary or less is missing a closing parenthesis: 73 (16.5%. Please correct.

Corrected.

Ensure consistency in the presentation of percentages — either include one decimal place throughout or none.

Corrected.

5. **Discussion:** The Discussion section appropriately interprets the study findings and situates them within existing research. However, it is overly verbose in several areas, with some repetition and dense technical explanation. The authors are encouraged to summarize model fit results more concisely, reconcile unexpected gender differences with prior findings, and expand on the limitations regarding psychometric properties. The clinical and research implications are important and well stated, but would benefit from tighter language. Overall, this section can be significantly improved with focused editing.

We made the changes to the discussion based on the reviewer’s comments. We hope it reads better now.

6. **Conclusions**
i. Overstatement of "Successful Validation"

Sentence rephrased:

This study **showed the psychometric properties** of the Arabic version of the SFLQ, confirming its reliability and validity as an effective instrument for assessing food literacy in Lebanon.

ii. Generalization beyond Study Sample: The conclusion appropriately acknowledges the need for broader validation. However, it would be useful to briefly reiterate the limitations of the sample (e.g., online-based, urban bias, higher education level) to ground the claim.
iii. Suggestion for Future Research

**Answer to the two previous comments:**

We added the following sentences to the conclusion:

**CONCLUSION**

This study showed the psychometric properties of the Arabic version of the SFLQ, confirming its reliability and validity as an effective instrument for assessing food literacy in Lebanon. **However, the sample was recruited primarily through online platforms, which may have led to an urban bias and an overrepresentation of individuals with higher education levels, thereby limiting the generalizability of the findings.** **Future research should extend the validation of this tool to a broader and more diverse Arabic-speaking population, including those with limited internet access, in rural settings and with various educational levels.** This would further enhance its generalizability and cultural relevance across the Arabic-speaking world.

**Thank you**

Thank you for your time, efforts and comments. We hope the revised version is up to your expectations.
